# Revitalizing tetanus vaccine literacy post road accidents: a comprehensive examination of knowledge and awareness initiative among non-medical university students in Lahore, Pakistan

**DOI:** 10.3389/fimmu.2024.1468356

**Published:** 2024-12-18

**Authors:** Fiza Ayub, Wahaj Saleem, Muhammad Awais Shahid, Muhammad Afzal Hussain Khan, Amal K. Suleiman, Laiba Akram, Sadaf Sajid, Allah Bukhsh, Mirza Rafi Baig, Tahir Mehmood Khan

**Affiliations:** ^1^ Institute of Pharmaceutical Sciences, University of Veterinary and Animal Sciences, Lahore, Punjab, Pakistan; ^2^ Department of Pharmacy Practice, College of Clinical Pharmacy, King Faisal University, Al-Ahsa, Saudi Arabia; ^3^ Department of Clinical Pharmacy and Pharmacotherapeutics, Dubai Pharmacy College for Girls, Dubai, United Arab Emirates

**Keywords:** tetanus vaccine, road accidents, tetanus knowledge, vaccination, tetanus, Pakistan

## Abstract

**Introduction:**

Tetanus, caused by *Clostridium tetani*, poses a life-threatening risk by affecting the nervous system and inducing muscle tightness. The objective of this study is to examine the knowledge, attitudes, and behaviors of non-medical university students regarding the tetanus vaccine in the context of post-road accidents.

**Methods:**

A descriptive cross-sectional study was conducted in 2023, involving 378 students from non-medical disciplines, primarily from information technology, business administration, and engineering faculties, with a mean age of 20.7 years. The sample size was calculated using the Raosoft sample calculator, and participants were selected via consecutive random sampling. Data was collected through a structured, self-administered questionnaire and analyzed using SPSS 22.0.

**Results:**

Gender distribution was 51.6% female and 48.4% male. Out of the 378 students, 53.4% (p=0.003) were unaware of tetanus contraction sources, 52.8% (p=0.004) lacked knowledge of its symptoms and 68.3% (p=0.004) were unfamiliar with the total number of doses. An appreciable 88.9% (p0.063) are willing to receive post-accident tetanus vaccination, reflecting positive attitudes and openness to preventive measures. Similarly, the p-values >0.05 show no gender disparity in willingness to receive tetanus vaccination (p=0.063) and recommending vaccination to others after a road accident (p=0.879).

**Conclusions:**

Notwithstanding participants' positive attitudes, the results indicate poor knowledge of tetanus regarding its transmission, symptoms, and doses. Interventions should focus on refining practical aspects of education, including symptom recognition, vaccine efficacy, duration, and the importance of sustaining immunity.

## 1 Introduction

Tetanus has been known since the time of Hippocrates. The condition used to be extremely deadly, with a high death rate. Modern therapeutic approaches and medical advancements have helped to lower the death rate, but it is still quite high (1). There is virtually little knowledge in the community on tetanus vaccination regimens and their significance in avoiding tetanus (2). A tetanus epidemic was documented in 2005 amid a significant earthquake in Pakistan’s north, with 23 recorded deaths. While tetanus still poses a danger of death in poor countries, its incidence has significantly decreased in the industrialized world. Consequently, there is a terrible need to increase public awareness and enhance the immunization program (3).

The precise incidence of tetanus among adult Pakistanis is unknown. Between 1995 and 1999, the Civil Hospital in Karachi received reports of over 100 adult cases of tetanus annually. Males made up 81% of these individuals (4).

Clostridium tetani is the cause of tetanus, a serious but non-contagious infectious disease. Wounds contaminated with dirt, dust, or excrement are the entry point for its spores. Other routes of infection include cuts, burns, or small scratches (5). Even though effective antibodies are now readily available as vaccines, tetanus infection continues to be a serious health concern in third-world countries. Because of the bacterium’s toxins, it is a potentially fatal non-communicable disease (6). Tetanospasmin is the tetanus-causing neurotoxin generated by vegetative cells of Clostridium tetani. With the help of peripheral nerves and retrograde axonal transport, this neurotoxin enters the central nervous system (CNS). It causes sustained excitatory discharge of disinherited alpha motor neurons, which in turn causes muscle stiffness and spasms (7).

Antitoxins are essential for neutralizing neurotoxins because they prevent them from entering the central nervous system (CNS). Decennial booster shots are advised for those who have received vaccinations to maintain protective levels. Maintaining immunity against neurotoxins is facilitated by the monitoring of antitoxin levels. Tetanus immune globulin (TIG), which provides instant passive immunity, and the tetanus toxoid vaccine, which provides active immunity, are the two options used to contrast tetanus neurotoxin action. Both are essential for treating and preventing tetanus.

The minimum protective level of antitoxin against tetanus is typically considered to be a serum concentration of at least 0.1 IU/mL. This level ensures immunity against neurotoxin.

Regular booster vaccinations every 10 years help to maintain this protective level of antitoxin and ensure continued immunity. Neurotoxins block nerve signals that travel from the brain to the spinal cord and subsequently to the muscles. This results in tetanus-related muscle spasms. Neonatal tetanus and generalized tetanus, the latter being the most common type, are the two types of tetanus (2). Due to high levels of ignorance and illiteracy, the disease is widespread in developing countries. Developed countries show low incidence due to immunization and awareness. In our country, incomplete immunization and low public awareness contribute. Other risk factors include unsterilized environments and septic abortions in peripheral areas caused by earthquakes or drought (1).

Tetanus is a devastating infectious disease that kills 10-80% of people (8). It causes symptoms such as convulsions, lockjaw, and severe muscular spasms, which can lead to spinal fractures and respiratory failure. Despite extensive immunization, the disease remains prevalent (6). Metronidazole or penicillin are used to treat symptoms, which can occur at any age and include fever, perspiration, and elevated blood pressure (9).

A 2015 retrospective research found 56,743 tetanus-related fatalities, including 19,937 in neonates and 36,806 in older children and adults (6). Tetanus predominantly affects persons in underdeveloped countries, where adult mortality rates are high, although cases in industrialized countries have dropped as a result of improved wound treatment, sanitary deliveries, and vaccines (10). In 2002, tetanus deaths overtook rabies mortality, with 198,000 children under the age of five. Pakistan, one of nine Asian countries battling to combat neonatal tetanus, has significant infant death rates (11). Grade 4 tetanus, which causes severe spasms, has a death rate of more than 50%, with risk factors including poor vaccination and concomitant illnesses (12). Key risks include car accidents, inadequate immunization, concurrent infections, burns, crushing injuries, and immunocompromised status due to a weakened immune system (7). Over the past ten years, Pakistan’s neonatal tetanus toxoid (TT) vaccination coverage has ranged from 60% to 74% (3).

The tetanus immunization regimen consists of three initial doses at ages 3, 5, and 11 months, two boosters at ages 6 and 11-15, and a booster every 10 years (5). If vaccination is no longer valid, a booster should be administered within 48 hours of damage (8). Many teenagers and adults are still exposed owing to missing boosters. Vaccines, such as DPT3 (diphtheria-pertussis-tetanus), are critical for public health, with 86% worldwide coverage in 2016, according to the WHO (10).

Pakistan, with 220 million inhabitants, faces challenges in healthcare. Infant mortality is high at 58 per 1,000 live births, and the annual growth rate is 2%. Since 1978, the Expanded Program on Immunization (EPI) has tackled diseases like tetanus and polio. However, Pakistan lags behind Bangladesh and Sri Lanka in immunization coverage, ranking third in unvaccinated children. Barriers include weak health infrastructure, limited accessibility, low education levels, and insufficient policy implementation. Key factors for low coverage are unmotivated EPI staff, lack of accountability, and limited private-sector involvement (13). As a result, there is a terrible need for the immunization program to be improved as well as for the general public to be made more aware of this deadly infection (3). Pakistan, a country with limited healthcare infrastructure, exhibits high tetanus prevalence, particularly following natural disasters or in settings where sterilization practices are inadequate​.

Pakistan’s Expanded Program on Immunization (EPI) advises that pregnant women have two doses of tetanus toxoid vaccination, ideally in the second and third trimesters. This immunization not only protects the mother but also distributes antibodies to the infant, giving critical early immunity (14, 15).

Adults should get a primary series of three tetanus toxoid doses, followed by a booster dose every ten years to maintain immunity. This provides continuing tetanus protection throughout adulthood (15, 16).

The EPI has made tremendous gains in lowering maternal and neonatal tetanus mortality in Pakistan. However, obstacles persist, such as poor immunization coverage in some areas and a need for greater public understanding of the need for vaccination (14, 15). Globally, tetanus remains a pressing health issue. According to recent estimates, the disease caused approximately 56,743 deaths in 2015, with a notable burden among neonates and older children in low-resource settings​. High rates of mortality persist in developing nations, where vaccination rates are lower and healthcare access is limited. However, the industrialized world has largely controlled tetanus through widespread immunization programs and robust post-exposure prophylaxis practices.

In terms of economic impact, tetanus contributes to the global healthcare burden due to its high costs of intensive care for severe cases and prolonged recovery times for survivors. Effective tetanus vaccination programs reduce mortality and lessen the economic strain on healthcare systems, especially in countries struggling to meet primary healthcare demands. The vaccine has proven to be one of the most cost-effective measures for public health, aligning with global goals to prevent avoidable diseases and reduce healthcare costs.

Given the high prevalence of road accidents in countries like Pakistan, awareness and accessibility of post-exposure tetanus prophylaxis are critical. This study seeks to explore the level of knowledge and attitudes towards tetanus vaccination among non-medical university students in Pakistan, an essential demographic due to their high exposure to traffic-related injuries. By assessing vaccination literacy and attitudes, we aim to identify gaps that can inform targeted public health interventions, improving vaccination uptake and disease prevention within this population segment.

Health professionals are crucial for immunization administration. Positive attitudes and s boost vaccine uptake. Doctors may lack tetanus prevention understanding. Students in frequent traffic accidents need education on infection seriousness and vaccination urgency (8). University students in non-health departments like engineering, BBA (bachelor of business administration), IT (information technology), and architecture lack awareness about disease risk factors, severity, mortality, vaccination, and post-exposure prophylaxis. To plan interventions effectively in Pakistan, understanding public perceptions, attitudes, and awareness about disease prevention and treatment is crucial (11).

This study aims to assess tetanus knowledge among non-health science students aged 18-25 post-road accidents. The goal of our study was to evaluate students understanding of the following aspects: post-exposure prophylaxis, vaccine availability, and affordability, significance of tetanus infections, particularly fatality rates, and risk factors for acquiring the disease. In particular, the study examined how age, gender, and educational attainment affect people’s knowledge (11). The high burden of tetanus infection in our society despite the availability of vaccines provides a strong justification for conducting this study. Despite higher vaccination rates, educated individuals often harbor anti-vaccination sentiments due to concerns about side effects and a lack of information. Limited research focuses on the vaccination attitudes of students in Pakistan, warranting further investigation (13). No population-based research has examined education’s impact on tetanus vaccination status. This study aims to identify demographic factors associated with lacking tetanus vaccination among adults over 18 in Pakistan (17).

We found a major gap in this study as most of the studies are conducted on health literacy and awareness of tetanus vaccination in women of childbearing age or pregnant women (18, 19) and vaccine literacy and awareness in medical students of Pakistan (20). Given the global prevalence of road accidents and their tetanus infection risk, understanding students’ awareness is crucial. Limited research exists on tetanus related to road accidents, especially in non-medical universities, leaving gaps in public health education. Non-medical students have varying health literacy levels, warranting targeted interventions to enhance vaccine literacy and tetanus immunization rates in this demographic. There is a difference in the knowledge of male and female students regarding tetanus vaccination.

This university-based, cross-sectional study was carried out to evaluate non-medical university students at Pakistani engineering and business institutions regarding their knowledge, attitudes, and behaviors regarding tetanus toxoid (TT) immunization, as well as Tetanus Vaccine Literacy Post Road Accidents. The primary goal of this study is to assess the knowledge, awareness, and attitude of students towards tetanus infection. This will lead to the identification of awareness gaps and, the designing, and implementation of awareness campaigns among students to improve their literacy about it. The results of this research will help in contributing to public health and educational strategies.

For an elusive picture following is a data comparing the amount of tetanus vaccination administered/year in Pakistan in **Table 1**.

**Table 1:** Comparison of the yearly administration of tetanus vaccinations in Pakistan.

Year	Total tetanus vaccination coverage (%)	Reported tetanus cases	Tetanus vaccine shortage in accident cases	Source
2019	~87% for DTP (Diphtheria, Tetanus, Pertussis)	High-risk in accident cases	The shortage led to high demand and a black market	WHO/UNICEF, Geo News
2020	~83% coverage, slight decrease	Limited supply, especially in high-demand scenarios like injuries	Issues in raw material supply led to shortages, especially in Karachi	UNICEF, Geo News
2024	Shortages ongoing in major cities	Increased demand for injured accident victims	Injection costs surged due to the shortage	Geo News, CDC

## 2 Material and method

### 2.1 Study design and population

A descriptive, cross-sectional, survey-based study was conducted in Lahore, Pakistan over a period from the 25^th^ of September 2023 to the 19^th^ of October 2023 (21–24).

### 2.2 Study setting

The questionnaire was disseminated in non-allied health departments i.e. information technology, engineering i.e. electrical, mechanical, civil department, agriculture, architecture, and bachelor of the following universities: University of Engineering and Technology (UET); University of Management and Technology (UMT); and Government College University Lahore (GCUL).

Inclusion criteria are non-medical and non-allied health science degree; Age 18-25; and Willingness to take part in the research. Exclusion criteria are Medical or allied health science degree; Age <18 or >25; Cognitive impairment; Language barrier; and Refusal to participate (22, 23).

### 2.3 Sample size

The target sample size was calculated using the Raosoft sample calculator. At the confidence level of 95%, the margin error was 5% and the P-value was assumed to be less than 0.05. Accordingly, a sample size of 378 respondents was required (23). A consecutive sampling technique was employed to recruit 378 students as respondents in this research.

### 2.4 Data collection

The study tool used was a specifically designed structured questionnaire. The questionnaire was validated and the reliability co-efficient was found to be 0.7. It had 16 questions which were closed-ended, and divided into 4 sections: Section A – Demographics (gender, age, department); Section B – Knowledge (contraction, symptoms, doses); Section C – Attitude (willingness to get vaccinated); and Section D – Recommendations (ways to improve knowledge) available as in supplementary file. The questionnaire was based on guidelines on tetanus vaccination provided by the Biological Production Division National Institute of Health Islamabad-Pakistan. The forms that were improperly filled in or had flaws were discarded. The data was collected by face-to-face interviews with participants. The questionnaires utilized during these interviews maintained participant anonymity. The data collection was continued until the target sample size was achieved (21–23).

### 2.5 Data analysis

The data generated from this research was entered and analyzed using Statistical Package for Social Sciences (SPSS) version 22.0 software. In SPSS, responses were coded as follows; Males as ‘1’, females as ‘2’, Yes as ‘1’, and no as ‘2’. The distribution of the data was examined using the Kolmogorov-Smirnov test, revealing the data was not normally distributed as the p-value was less than 0.05. Additionally, Skewness and Kurtosis analyses further confirmed that the data is not normally distributed. For data analysis, the statistical significance level was 0.05 with a confidence interval of 95%. The data were evaluated using parametric statistics such as frequencies, mean, median, standard deviation, and cross-tabulation. To explore the association between respondents’ socio-demographic characteristics and their practice of toxoid immunization, the Chi-square test was utilized.

### 2.6 Ethical consideration

The IRB Committee of the University of Veterinary and Animal Sciences, Lahore reviewed and approved the study. A consent form that had the title and the purpose of the study was attached as the first page of the questionnaire. Only those who voluntarily agreed to participate were included in the research.

## 3 Results

### 3.1 Demographics

The predominant demographic in our study comprised individuals below the age of 25. The mean age was 20.70± 1.747 years. All participants were enrolled as university students, with 51.6% being female and 48.4% male.

Notably, a significant portion of the participants 81.41% had not undergone any first-aid training. All students belonged to non-medical and non-health sciences disciplines, With the highest representation from the IT department (53.4%) (**Table 2**).

**Table 2:** Demographics of respondents (N=378).

Demographics characteristics			
Variable		Frequency	Percentage
Gender			
	Male	183	48.4%
	Female	195	51.6%
Age			
	18-21 Years	267	70.6%
	22-25 Years	111	29.4%
Degree			
	BBA	47	12.4%
	Architecture	32	8.5%
	Engineering	97	25.7%
	IT	202	53.4%

IT, Information Technology; BBA, Bachelor of Business Administration.

### 3.2 Knowledge of respondents

Approximately 53.4% of the students lacked awareness regarding the contraction of tetanus, and 52.8% were unfamiliar with the Associated Regarding symptoms. Regarding tetanus vaccination, 65.6% of students were aware of the recommended timeframe for vaccination post-accident. However, 87.0% did not know whether prophylaxis was necessary if the vaccination had been administered within a year of the present injury. Additionally, 68.3% were unaware of student’s the total number of tetanus vaccines required. Women demonstrated a significantly higher knowledge level than men (p = 0.003) (**Table 3**).

**Table 3:** Knowledge of participants about tetanus and its vaccine.

Knowledge				
Variable		Frequency (%)		p-value
Do you know how tetanus is contracted?		Yes	No	
	**Male**	71 (38.8%)	112 (61.2%)	
	**Female**	105 (53.8%)	90 (46.2%)	
	**Total**	176 (46.6%)	202 (53.4%)	**0.003**
Are you aware of the symptoms of tetanus?				
	**Male**	70 (38.3%)	113 (61.7%)	
	**Female**	103 (57.6%)	92 (47.2%)	
	**Total**	173 (45.8%)	205 (52.8%)	**0.004**
Do you know when a tetanus vaccine is typically recommended after an injury or accident?				
	**Male**	134 (73.2.8%)	49 (26.8%)	
	**Female**	114 (58.5%)	81 (41.5%)	
	**Total**	248 (65.6%)	130 (34.4%)	**0.003**
Have you received a tetanus vaccination in the past 5 years?				
	**Male**	100 (54.6%)	83 (45.4%)	
	**Female**	52 (26.7%)	143 (73.3%)	
	**Total**	152 (40.2%)	226 (59.8%)	**0.001**
If tetanus vaccination was performed one year or less before the present injury, is prophylaxis necessary?				
	**Male**	33 (18.0%)	150 (82.0%)	
	**Female**	16 (8.2%)	178 (91.8%)	
	**Total**	49 (13.0%)	329 (87.0%)	**0.004**
Do you know the total number of doses of tetanus vaccination?				
	**Male**	71 (38.8%)	112 (61.2%)	
	**Female**	49 (25.1%)	146 (74.9%)	
	**Total**	120 (31.7%)	258 (68.3%)	**0.004**

A chi-square test was applied. The p< 0.05 was considered significant. The bold p-value indicates statistical significance, as all values are less than 0.05, demonstrating a significant relationship between the respondents and their knowledge of tetanus and its vaccine.

### 3.3 An attitude of participants towards tetanus vaccination

Despite knowledge gaps, 88.9% expressed willingness to receive a tetanus vaccination if recommended post-accident and 82.3% had recommended the vaccine to someone involved in an accident. No statistically significant difference was found between male and female students’ attitudes toward tetanus vaccination (p > 0.05) (**Table 4**).

**Table 4:** Attitude of participants towards tetanus vaccination.

Attitude				
Variable		Frequency (%)		p-value
Do you believe tetanus vaccinations are important for preventing infections after road accidents?		Yes	No	
	Male	131 (71.6%)	52 (28.4%)	
	Female	151 (77.4%)	44 (22.6%)	
	Total	282 (74.6%)	96 (25.4%)	0.192
Would you be willing to get a tetanus vaccination after a road accident if recommended?				
	Male	157 (85.8%)	26 (14.2%)	
	Female	179 (91.8%)	16 (8.2%)	
	Total	336 (88.9%)	43 (10.8%)	0.063
Have you ever recommended tetanus vaccination to someone involved in a road accident?				
	Male	150 (82.0%)	33 (18.0%)	
	Female	161 (82.6%)	34 (17.4%)	
	Total	311 (82.3%)	67 (17.7%)	0.879

A chi-square test was applied. The p< 0.05 was considered significant.

### 3.4 Recommendations of participants

A majority 88.1% advocated for including tetanus vaccination information in driver education programs. Additionally, 80.2% supported online platforms and social media for tetanus vaccine awareness, and 84.4% recommended a standardized information booklet or pamphlet on tetanus vaccination for university students. Responses differed significantly between males and females on integrating vaccination information into driver programs (p = 0.048). Although no significant gender differences were observed regarding the effectiveness of online platforms (p = 0.341), the overall inclination toward these mediums was positive. There was a highly significant difference between male and female opinions on the need for a standardized information booklet (p < 0.001) (**Table 5**).

**Table 5:** Recommendations of participants.

Recommendations				
Variable		Frequency (%)		p-value
Would you recommend integrating tetanus vaccination information into driver programs?		Yes	No	
	**Male**	155 (84.7%)	28 (15.3%)	
	**Female**	178 (91.3%)	17 (8.7%)	
	**Total**	333 (88.1%)	45 (11.9%)	**0.048**
Do you think online platforms and social media are effective for spreading tetanus vaccine awareness?				
	**Male**	143 (78.1%)	40 (21.9%)	
	**Female**	160 (82.1%)	35 (17.9%)	
	**Total**	303 (80.2%)	75 (19.8%)	0.341
Should there be a standardized information booklet or pamphlet on tetanus vaccination distributed to all				
**University students?**	**Male**	142 (77.6%)	41 (22.4%)	
	**Female**	177 (90.8%)	18 (9.2%)	
	**Total**	319 (84.4%)	59 (15.4%)	**<0.001**

A chi-square test was applied. The p< 0.05 was considered significant. The bold p-value indicates statistical significance, as it is less than 0.05. This significance is observed between respondents' opinions on integrating tetanus vaccination information into driver programs and the need for a standardized information booklet. However, no significant gender differences were found regarding the effectiveness of online platforms.

## 4 Discussion

Tetanus is a life-threatening bacterial infection that affects the nervous system, causing muscle tightness and spasms. This can lead to a lockjaw, where the muscles in the jaw and neck become rigid. If left untreated, tetanus can be fatal. This study aimed to assess the knowledge about tetanus vaccine post-road accidents among university students. A descriptive, cross-sectional study design was used to implement this study. The findings of our study revealed that a significant proportion of the participants were cognizant of both the presence of tetanus infection and the availability of the tetanus toxoid vaccine. This aligns with observations from prior studies, wherein over half (55.3%) of the participants demonstrated awareness of tetanus disease. The congruence between our study’s outcomes and those of earlier research underscores the consistency and prevalence of knowledge regarding tetanus infection and the corresponding preventive vaccination across diverse study populations (25).

It is noteworthy to highlight that nearly half (53.4%) of the students displayed a lack of knowledge concerning the mode of tetanus contraction. This finding aligns with the results of a preceding study, which similarly reported that 46.8% of their participants lacked awareness regarding the means of tetanus transmission. The consistency in these findings underscores the persistent gap in understanding among students regarding how tetanus is contracted (25). The findings of the study unveiled a significant lack of awareness among most participants, with 87.0% expressing uncertainty regarding the necessity of prophylaxis within one year or less following tetanus vaccination in the event of a current injury. This outcome aligns with a prior investigation that highlighted a similar pattern within the general public, where a mere 3.1% demonstrated knowledge about the prophylaxis of tetanus in adults (26).

A point of significance is the observation that a substantial 68.3% of the respondents lacked awareness regarding the total number of tetanus vaccine doses required following an injury. This stands in stark contrast to a separate study conducted in India, where nearly 0% of the general public possessed knowledge about the requisite number of tetanus doses after an injury. The evident disparity in awareness between university students and the general public underscores the impact of varied knowledge levels within distinct demographic groups (26). The predominant portion of participants in our study, constituting 75.3%, affirmed the availability of tetanus vaccination in Pakistan. This figure aligns with findings from a comparable study, where 53.2% of participants were aware of the existence of tetanus prevention vaccines in Pakistan (25). The outcome of patients with tetanus has been reported variably & is significantly affected by many factors which include age grade of presentation, development of complications, presence of co-morbid factors & presence of effective tools of treatment (27). Literature reports mortality from tetanus in the adult population ranges between 10-50% which in neonates approaches 90-95% (28).

Despite the availability of the tetanus vaccine, there remains a significant gap in its complete implementation. This gap is attributed to a lack of awareness and understanding among the population regarding the importance of tetanus vaccination, particularly following injuries, or accidents. There exists a disparity in the understanding of tetanus vaccination between males and females, with males demonstrating a higher level of knowledge on the subject. Many individuals fail to receive the requisite number of doses or adhere to the recommended booster schedule, leaving them vulnerable to tetanus infection (29).

To address this issue and promote tetanus prevention, it is essential to raise public awareness and educate individuals about the disease’s severity, the effectiveness of the vaccine, and the importance of completing the vaccination schedule. Additionally, healthcare providers should play a crucial role in counseling patients and reinforcing the importance of tetanus vaccination, particularly following injuries, or accidents. By enhancing knowledge and promoting adherence to vaccination protocols, we can effectively prevent tetanus and its associated complications.

### 4.1 Limitations

This study sheds light on tetanus knowledge, attitudes, and behaviors among non-medical university students after road accidents, but limitations call for careful interpretation. Its strengths like timeliness, targeted focus, and adequate sample size provide valuable data for targeted interventions. However, its cross-sectional design and limited demographic scope restrict causal inferences and generalizability. Another limitation of our study is that vaccination coverage data were self-reported by individuals, which might have resulted in potential biases underestimated or exaggerated vaccination rates. However, self-reported, questionnaire-based data appear to be an appropriate strategy in similar research evaluating vaccination coverage (30, 31).

While positive vaccination attitudes offer a promising foundation, knowledge gaps in transmission and symptoms highlight areas for improvement. Integrating vaccination into driver education and leveraging online platforms, as suggested by the study, holds the potential for tailored strategies. Overall, the study lays the groundwork for further research to gain a more comprehensive understanding and develop effective tetanus prevention strategies for diverse populations.

## 5 Conclusion

University students exhibit concerning gaps in their understanding of tetanus, particularly regarding transmission, symptoms, and the number of doses of vaccine. This lack of knowledge emphasizes the urgency of implementing focused educational efforts to ensure their well-being. This necessitates interventions that refine specific aspects of tetanus education, focusing on practical information like recognizing symptoms, understanding vaccine efficacy and duration, and the importance of maintaining immunity. The positive attitude towards vaccination and openness to innovative educational strategies, like integrating with driver education and utilizing online platforms, offer promising avenues for targeted campaigns. By leveraging existing awareness and tailoring interventions, we can effectively enhance tetanus knowledge and preventive practices among university students, safeguarding their health and well-being.

## Data Availability

The original contributions presented in the study are included in the article/supplementary material. Further inquiries can be directed to the corresponding author.

## References

[1] Ayyub A Malik YJ Sarwar I. To determine influence of various risk factors of tetanus on its outcome. Asian J Multidiscip Stud. (2018) 6:6.

[2] Dahiya N Kumar R Meena GS Goel P. Knowledge and practices regarding the prevention of tetanus among adults of resettlement colony of Delhi, India. Indian J Med Specialities. (2019) 10:155–8. doi: 10.4103/INJMS.INJMS_36_19

[3] Naz A Rasheed G Rao ZA Urooj S Ismail H Kumar V. Clinical profile of adult tetanus patients and the impact of early tracheostomy on the outcome; a retrospective observational study. Anaesthesia Pain Intensive Care. (2021) 25:625–32. doi: 10.35975/apic.v25i5.1625

[4] Ahmed S Baig L Thaver I Siddiqui M Jafery S Javed A. Knowledge, attitudes and practices of general practitioners in Karachi District Central about tetanus immunization in adults. Journal-Pakistan Med Assoc. (2001) 51:367–9. 11768940

[5] Borella-Venturini M Frasson C Paluan F De Nuzzo D Di Masi G Giraldo M. Tetanus vaccination, antibody persistence and decennial booster: a serosurvey of university students and at-risk workers. Epidemiol Infect. (2017) 145:1757–62. doi: 10.1017/S0950268817000516 PMC9203330 28294099

[6] Abah MG Asuquo OA Inyangetoh EC. Knowledge and behaviour towards tetanus toxoid immunisation in south-south, Nigeria: findings from antenatal clinic attendees. Asian J Appl Sci. (2019) 7. doi: 10.24203/ajas.v7i5.5785

[7] Waqar K Riaz SU Ali A Fatima M Ishaq M Sohail H. Tetanus outcomes in atertiary care hospital in karachi. J Peoples Univ Med Health Sci Nawabshah(JPUMHS). (2021) 11:48–51. doi: 10.46536/jpumhs/2021/11.02.291

[8] Liu Y Mo X Yu X Wang J Tian J Kuang J. Insufficient knowledge and inappropriate practices of emergency doctors towards tetanus prevention in trauma patients: a pilot survey. Hum Vaccines Immunotherapeut. (2020) 16:349–57. doi: 10.1080/21645515.2019.1653745 PMC7062443 31625792

[9] Qadir MI Komal T. Public awareness about tetanus. J Sci Tech Res. (2019). doi: 10.26717/BJSTR.2019.14.002581

[10] Siddiqui AA Khan M Khan JA Haseeb SS Mohib A Kadri HM. Awareness, knowledge, and coverage of vaccination against tetanus, diphtheria, and pertussis among medical students of Karachi: a cross-sectional analysis. Cureus. (2019) 11. doi: 10.7759/cureus.4472 PMC6579328 31249750

[11] Wasay M Malik A Fahim A Yousuf A Chawla R Daniel H. Knowledge and attitudes about tetanus and rabies: a population-based survey from Karachi, Pakistan. J Pakistan Med Assoc. (2012) 62:378. Available at: https://ecommons.aku.edu/pakistan_fhs_mc_med_neurol/3 22755284

[12] Afshar M Raju M Ansell D Bleck T. Narrative review: tetanus-A health threat after natural disasters in developing countries. Ann Internal Med. (2011) 154:329–35. doi: 10.7326/0003-4819-154-5-201103010-00007 21357910

[13] Majeed A Hussain I Ashraf W Rehman AU Ahsan A Hamid S. Assessment of immunization status and barriers to vaccination among the university students of Pakistan. Vaccimonitor. (2021) 30:115–24.

[14] ISLAMABAD-PAKISTAN BPDNIOH. Tetanus Toxoid Adsorbed National Institute of Health Pakistan (2018).

[15] WHO. World Health Organization . Available online at: https://www.emro.who.int/pak/programmes/expanded-programme-on-immunization.html.

[16] FDI. Federal Directorate of Immunization . Available online at: https://www.epi.gov.pk/vaccine-preventable-diseases/tetanus/.

[17] Rencken CA Dunsiger S Gjelsvik A Amanullah S. Higher education associated with better national tetanus vaccination coverage: A population-based assessment. Prev Med. (2020) 134:106063. doi: 10.1016/j.ypmed.2020.106063 32197975

[18] Hashami MF. Awareness of Tetanus Toxoid vaccination among pregnant women attending Antenatal Clinic in Districts: Lahore &Sheikhupura-Pakistan. GSJ. (2020) 8.

[19] Humayun S Azam N. Awareness and uptake of tetanus toxoid vaccination among females of reproductive age in rawalpindi. Pakistan Armed Forces Med J. (2023) 73:506–09. doi: 10.51253/pafmj.v73i2.8404

[20] Riaz S Arif M Daud S. Immunization in medical students: Knowledge and practice. Pak J Med Health Sci. (2017) 11:1501–4.

[21] Salih BB Ahmed HAJ. Students’ Knowledge toward importance of tetanus toxoid vaccine for women and their children in al-bayan university. HIV Nursing. (2023) 23:1119–24–24.

[22] Alghamdi R Hassan Tayyib N. Knowledge, attitude, and awareness on (tetanus, diphtheria and acellular pertussis) vaccination during pregnancy in Kingdom of Saudi Arabia. Nurs Commun. (2023) 7:e2023022. doi: 10.53388/IN2023022

[23] Doğan A Mohamed Alİ A Abdullahi Alİ M Orul H. Assessment of tetanus Immunization among healthcare workers in Mogadishu, Somalia. Hum Vaccines Immunotherapeut. (2023) 2202128. doi: 10.1080/21645515.2023.2202128 PMC10294765 37133877

[24] Bint e Ajmal K Azam N Pervaiz F Akhtar SS Mahmood H Yousaf S. Knowledge attitude and practices regarding tetanus toxoid vaccination in reproductive age women (15-49). a descriptive crosssectional study in pak emirates military hospital, rawalpindi. Pakistan Armed Forces Med J. (2019) 69:S334–39.

[25] Haider A Manzoor I Hassan HB Samad A Fatima A Shahid B. Tetanus toxoid vaccination coverage and reasons for non-vaccination among married women in reproductive age group of 15—49 years. J Akhtar Saeed Med Dental Coll. (2019).

[26] Dabas P Agarwal C Kumar R Taneja D Ingle G Saha R. Knowledge of general public and health professionals about tetanus immunization. Indian J Pediatrics. (2005) 72:1035–7. doi: 10.1007/BF02724406 16388152

[27] Chalya PL Mabula JB Dass RM Mbelenge N Mshana SE Gilyoma JM. Ten-year experiences with Tetanus at a Tertiary hospital in Northwestern Tanzania: A retrospective review of 102 cases. World J Emergency Surgery. (2011) 6:1–8. doi: 10.1186/1749-7922-6-20 PMC3159100 21740539

[28] Sanya E Taiwo S Olarinoye J Aje A Daramola O Ogunniyi A. A 12-year review of cases of adult tetanus managed at the University College Hospital, Ibadan, Nigeria. Trop Doctor. (2007) 37:170–3. doi: 10.1258/004947507781524601 17716509

[29] Talpur AA Surahio AR Memon AS Junejo A Jamal A. Tetanus situation in Pakistan: comparison of medical versus surgical management. Prof Med J. (2016) 23:634–40. doi: 10.29309/TPMJ/2016.23.06.1598

[30] Avramidis I Pagkozidis I Domeyer P-RJ Papazisis G Tirodimos I Dardavesis T. Exploring perceptions and practices regarding adult vaccination against seasonal influenza, tetanus, pneumococcal disease, herpes zoster and COVID-19: A mixed-methods study in Greece. Vaccines. (2024) 12:80. doi: 10.3390/vaccines12010080 38250893 PMC10818817

[31] Maltezou HC Tseroni M Drositis I Gamaletsou MN Koukou DM Bolikas E. Vaccination coverage rates and attitudes towards mandatory vaccinations among healthcare personnel in tertiary-care hospitals in Greece. Expert Rev Vaccines. (2022) 21:853–9. doi: 10.1080/14760584.2022.2063118 35382665

